# Individuation of objects and object parts rely on the same neuronal mechanism

**DOI:** 10.1038/srep38434

**Published:** 2016-12-07

**Authors:** Marlene Poncet, Alfonso Caramazza, Veronica Mazza

**Affiliations:** 1 Center for Mind/Brain Sciences (CIMeC), University of Trento, Italy; 2 Department of Psychology, Harvard University, Cambridge, MA, USA.

## Abstract

Recent results have shown that participants can enumerate multiple parts of a single object as efficiently as multiple distinct objects, suggesting a shared mechanism for individuation of objects and object parts. Here we used the subitizing phenomenon to investigate the neural mechanism underlying the individuation of object parts. In two experiments, we measured a lateralized EEG response (N2pc) previously associated with individuation of multiple objects. In line with the subitizing effect, participants’ error rate was low (less than 10%) when enumerating up to approximately three parts of an object but increased for larger numerosities. The N2pc amplitude increased as a function of the number of object parts, and reached an asymptote corresponding to the subitizing limit, replicating previous reports for separate objects. These results invite the inference that the same neural mechanism underlies individuation of multiple distinct objects and multiple parts of a single object.

The ability to individuate multiple objects efficiently has been the focus of intensive research in several areas of cognitive neuroscience. One way to study the mechanisms underlying this ability is to use enumeration tasks, in which participants report the number of target elements presented in cluttered (i.e., among distractors) or uncluttered (i.e., in isolation) scenes. Fast and accurate responses are typically found for up to ~4 target elements, a phenomenon known as “subitizing”^1^. In contrast, participants’ error rates and/or reaction times increase more steeply for each item added beyond this ~4 objects limit. This results in an elbow-like pattern of performance, with a shallow enumeration slope up to ~4 items and a steep enumeration slope above 4 items^2^,^3^,^4^.

Two theoretical accounts have been proposed for the subitizing effect (see ref. 5 for a recent review). According to the “estimation” account, subitizing is the result of the operation of an “approximate” system. This system codes for numerosity by providing an analog representation, as in the case of other sensory stimulus dimensions^6^. By contrast, other models^7^ have assumed that subitizing reflects the operation of an individuation mechanism that binds the information about an object to its location^1^,^8^,^9^ (but see refs 10, 11, 12). This mechanism has a limited capacity, such that only ~4 objects can be tagged to their respective locations simultaneously. While the debate between these two positions is still open, recent studies have shown that subitizing may be different in nature from estimation. For example, the two processes do not require the same attentional resources^13^,^14^,^15^ and individual differences in subitizing capacity do not correlate with individual differences in large number estimation precision^16^. In addition, subitizing capacity correlates with visual-working memory capacity during delayed match-to-sample judgements, another task that requires processing and individuating a small number of objects simultaneously^16^. Taken together these results suggest that small numerosities are processed by a capacity-limited multiple object individuation mechanism.

Individuation of multiple objects has been studied using an EEG marker of the direction of attention: the N2pc^17^. This component, appearing around 200 ms after stimulus onset in the parieto-occipital electrodes, is defined as the difference between the activities of the electrodes contralateral versus ipsilateral to the target side. In enumeration tasks (as well as in other tasks requiring multiple target individuation, such as multiple object tracking^18^), N2pc increases with the number of objects to be enumerated, and reaches an asymptote at approximately 3-4 elements. This ~4 elements limit corresponds to the point at which participants are no longer able to efficiently enumerate the objects^19^,^20^. In addition, individual differences in N2pc correlate with individual differences in the subitizing range^21^,^22^. On the basis of these results, Mazza and Caramazza^23^ proposed that the N2pc modulation related to the number of target objects to be enumerated and its asymptote at ~4 elements reflect the operation of a capacity-limited, attention-based individuation mechanism.

Efficient individuation is assumed to function on distinct objects. Previous studies suggested that this mechanism would only operate on object representations and not on object features^24^,^25^. However, the results of a recent study contradict this view. Porter *et al*.^26^ investigated whether features of an object, such as parts, could be individuated simultaneously. In their study, the elements to be individuated occupied spatially distinct locations (just as multiple objects would do) but were all bound to the same object, and as such presumably belonged to the same object representation. Their results showed that participants could enumerate a small number of object parts (up to ~4) very efficiently (in terms of speed and accuracy). Participants’ performance for object parts was essentially indistinguishable from that observed for the enumeration of multiple distinct objects. The authors concluded that simultaneous individuation operates over both distinct objects and object parts.

Given that Porter *et al*.^26^ used only behavioural measures, it was not possible to determine if the individuation of multiple parts of an object relied on the same or on different neuronal mechanisms compared to the individuation of distinct objects. In the present EEG study we examined whether the N2pc is modulated by object-part numerosity in the same way as has been found for distinct objects numerosities. We addressed this question in two experiments in which we recorded participants’ EEG while they enumerated parts of an object. In Experiment 1, we presented two solid half discs with outdents in each hemifield. Participants had to enumerate the number of outdents of one coloured object and ignore the object on the other side of the screen. The goal of Experiment 2 was to strengthen and generalize our findings by using other experimental contexts (for a similar logic see ref. 26). To this end, in Experiment 2 we changed the target shapes (we used indents instead of outdents), and presented only a single object with multiple parts. Participants were asked to report the number of parts of one side of the object while ignoring the parts located on the other side of the same object. The cue was indicated by the background colour instead of the colour of the stimulus as in Experiment 1 (see Fig. 1 and Method section for more details). If the same mechanism underlies the simultaneous individuation of both objects and object parts (irrespective of the type of parts, the cue and the object used as a stimulus), we expected that in both Experiment 1 and Experiment 2, N2pc amplitude should be modulated by the number of object parts within the subitizing range but not by larger numerosities.

## Results

### Behavioural results

**Figure 1:**
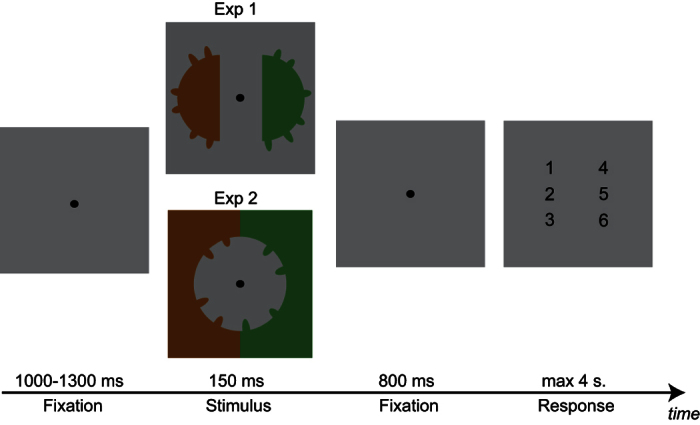
Illustration of a trial in experiments 1 and 2. Participants were asked to report the number of parts (outdents in Experiment 1, indents in Experiment 2) on the side cued by a colour indicated by the object in Experiment 1 (Exp 1) or by the background in Experiment 2 (Exp 2) by clicking with a mouse on one of the 6 digits presented at the end of the trial. For illustration purposes, the scale of the stimuli has been modified.

**Figure 2:**
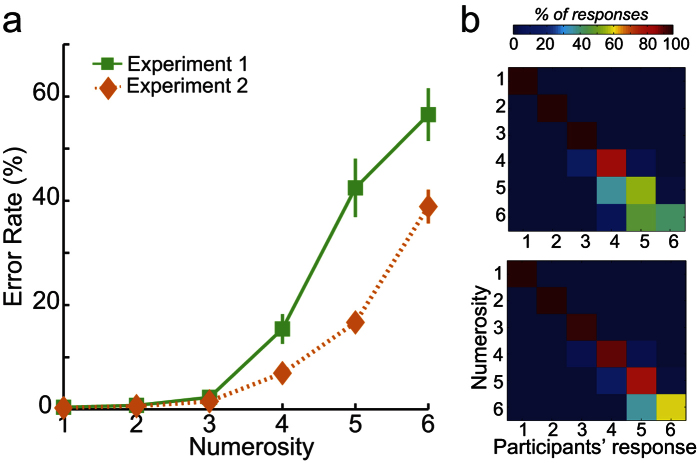
Participants’ behavioural performance. (**a**) Mean Error Rate as a function of the number of parts to be enumerated in experiments 1 (green squares) and 2 (orange diamonds). Error bars represent the SEM. (**b**) Confusion matrix representing participants’ responses at each numerosity in Experiment 1 (top) and Experiment 2 (bottom).

We assessed performance using error rates (ER), and not reaction times since our paradigm involved a delayed response (see Fig. 1). In Experiment 1, ER was less than 10% when participants had to report 1, 2 or 3 object parts but increased dramatically when participants had to enumerate 4, 5 or 6 parts (see Fig. 2a). This pattern of results suggests a subitizing effect for approximately up to 3 object parts. For large numerosities, participants underestimated the number of parts to be enumerated. For example, when 5 outdents were presented, participants reported perceiving 4 outdents in 40% of the trials and 5 in 60% of the trials (see Fig. 2b). We calculated a subitizing span (see Method) – that is, the number of parts that can be individuated simultaneously – using the exponential fit method (2.97 ± 0.26 *MEAN* ±*SEM* object parts; adjusted r^2^ = 0.89 ± 0.02) and the bilinear fit method (3.25 ± 0.13; adjusted r^2^ = 0.91 ± 0.02). On average, participants could efficiently enumerate 3 parts of the object.

In Experiment 2, participants’ ER was less than 10% for reporting 1, 2, 3 or 4 target parts but increased when participants had to enumerate 5 or 6 parts (Fig. 2a). As in Experiment 1, participants underestimated large numerosities (Fig. 2b). We found an estimation of the subitizing point at 4.24 ± 0.19 parts for the exponential fit (adjusted r^2^ = 0.96 ± 0.02) and 3.85 ± 0.20 for the bilinear fit (adjusted r^2^ = 0.93 ± 0.03). That is, participants could efficiently enumerate approximately 4 parts of the object.

### EEG results

#### N2pc

We calculated the lateralized response, the N2pc, using a cluster of parieto-occipital electrodes, in the time-window from 180 ms to 250 ms post-stimulus onset. The mean N2pc amplitude values were analysed via a repeated-measures (RM) ANOVA with numerosity (1–5) as main factor (see Methods for more details). We report here the results for EEG data pre-processed with the visual artefact rejection procedure. Analyses using the automatic artefact rejection procedure gave similar results (see Supplementary Data).

**Figure 3:**
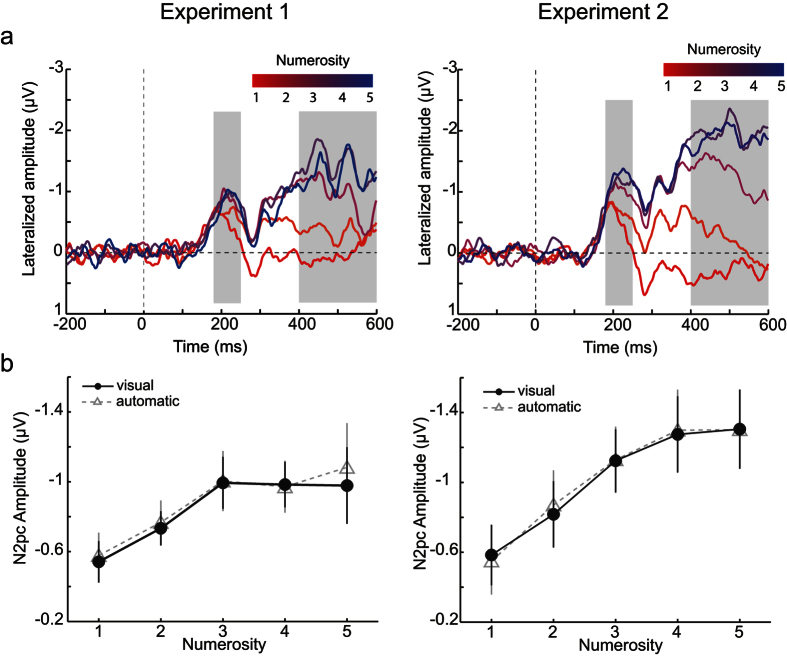
EEG results of Experiment 1 (left column) and Experiment 2 (right column). (**a**) Time course of the lateralized EEG signal for each target numerosity (from 1 in red to 5 in blue) after visual artefact rejection. The time ranges used for the N2pc (180–250 ms) and the CDA (400–600 ms) are indicated by a grey background. (**b**) N2pc mean amplitude for each numerosity after visual (black circles) or automatic (grey triangles) artefact rejection. Error bars represent the SEM.

The RM-ANOVA on the N2pc amplitude (Fig. 3, left column) showed a significant main effect of numerosity (F(2.16,28.05) = 3.93, p = 0.03, _p_η^2^ = 0.23) in Experiment 1. Post-hoc analyses, conducted using Helmert contrasts, indicated that when a 1-part object was presented, N2pc amplitude was lower than the mean amplitude for objects with more parts (F(1,13) = 10.69, p = 0.006, _p_η^2^ = 0.45). Similarly, when a 2-parts object was presented, N2pc amplitude was lower than the mean amplitude of higher numerosities (F(1,13) = 9.82, p = 0.008, _p_η^2^ = 0.43). In contrast, no difference was found between 3- vs. 4- and 5-parts objects, and between 4- and 5-parts stimuli (p > 0.9 for these two contrasts). To summarize, N2pc amplitude increased between 1 and 3 parts to be enumerated. It then stabilized and no amplitude difference was observed for 3, 4 and 5 parts.

In Experiment 2, N2pc amplitude (Fig. 3, right column) varied with the number of parts that were presented (F(4,52) = 14.17, p = 7 × 10^−8^, _p_η^2^ = 0.52). Helmert contrasts showed that N2pc increased with the number of object parts: N2pc amplitude was smaller for 1-part stimuli compared to higher numerosities (F(1,13) = 30.57, p = 0.0001, _p_η^2^ = 0.70), for 2-parts objects compared to higher numerosities (F(1,13) = 18.48, p = 0.001, _p_η^2^ = 0.59), and close to significance when comparing the N2pc amplitude for 3-parts objects to the mean N2pc amplitude for 4- and 5-parts stimuli (F(1,13) = 3.87, p = 0.07, _p_η^2^ = 0.23). There was no significant difference in N2pc amplitude between 4 and 5 parts (F(1,13) = 0.06, p = 0.81). Thus, N2pc increased with the increase in number of parts up to approximately 4 target elements.

#### CDA

As described in the introduction, the focus of the study was on the N2pc response. However, several studies on multiple object processing have indicated that the numerosity-related modulation of N2pc for multiple disconnected objects is often followed by a subsequent lateralized EEG modulation called Contralateral Delay Activity (CDA, also referred to as Sustained Posterior Contralateral Negativity^20^). This lateralized component appears around 300–400 ms after stimulus onset and was originally measured in memory tasks during active maintenance of multiple target elements^27^,^28^. Like N2pc, CDA amplitudes are modulated by the number of separate elements within the subitizing range in multiple object tracking^18^ and enumeration tasks^22^. However, the CDA modulation in the latter task is not strictly linked to the behavioural subitizing limit^20^,^22^. Thus, in the case of multiple separate objects it is the limit in simultaneous individuation (as reflected by the N2pc amplitudes) that ultimately determines the subitizing limit. Nonetheless, for completeness we also report the analysis on CDA.

We computed the CDA in the time-window from 400 ms to 600 ms after stimulus onset, using the same method and electrode cluster as for the N2pc. The RM-ANOVA applied on the CDA amplitudes in Experiment 1 indicated a significant effect of numerosity (F(1.81,23.49) = 24.41, p = 3 × 10^−6^, _p_η^2^ = 0.65). Helmert contrasts showed that the CDA amplitude increased significantly from 1- to 4-parts objects (p < 0.005 for each comparison). There was no significant difference between the CDA amplitudes at 4 vs. 5 parts (F(1,13) = 0.02, p = 0.89). Thus, CDA amplitude increased up to 4 object parts before reaching a plateau.

In Experiment 2, CDA amplitude was also affected by the number of object parts (F(2.29,29.79) = 65.69, p = 3 × 10^−12^, _p_η^2^ = 0.83). Helmert contrasts were significant from 1 to 4 object parts (p < 0.0005 for each contrast) but we found no difference between the CDA amplitudes when 4- or 5-parts objects were presented (F(1,13) = 0.28, p = 0.60). Thus, CDA amplitude increased up to 4 object parts before stabilizing.

## Discussion

An important issue in the field of perception and attention concerns the units of individuation. Influential theories of vision (e.g.ref. 7) argue that individuation operates over separate entities; such entities are typically assumed to be “objects”, intended as physically disconnected elements. In line with this observation, seminal research on infants has identified spatiotemporal cues as the primary source of information used by infants to individuate objects^29^,^30^, whereas the distinction based on other non-spatial properties of objects occurs only later^31^. Converging findings in human adults seem to support this idea. For instance, no subitizing phenomenon emerges when enumerating non-spatial features, such as the number of colours in a multiple items display^25^. Thus, spatial distinctiveness seems to be an important factor in the emergence of subitizing, and “objects” are typically considered the spatially distinct unities of individuation. However, “spatially distinct” and “object” are not synonymous, raising the question of whether spatial distinctiveness is sufficient to support individuation of items that are parts of the same object. A recent behavioural study by Porter *et al*.^26^ found that efficient individuation can occur over objects as well as over object parts, as long as they occupy distinct locations in space. In several experiments, the authors found a subitizing effect both when participants had to enumerate the number of distinct objects and when they had to report the number of multiple parts connected into a single object. These results challenge the common view that individuation equals “distinct object individuation”.

In the present study, we investigated whether individuation of multiple parts of a single object (namely, spatially distinct entities that are connected to one another) involves the same or different neural mechanisms as those for multiple objects (defined as spatio-temporal entities that are disconnected from one another). Finding equivalent results for object parts and distinct objects would strengthen the view that the representations over which individuation operates can flexibly switch from physically distinct objects to connected parts of an object occupying separate locations in space.

The behavioural results of both Experiment 1 and Experiment 2 showed efficient enumeration for a small number of parts (approximately 3-4 target elements), with fewer than 10% errors. In contrast, larger target numerosities induced a larger number of errors. The overall pattern of error rates in both experiments indicated the presence of a typical subitizing effect, with a shallow slope for the enumeration of a small number of object parts, and a steeper slope for larger object-parts numerosities. These results replicate behavioural studies on enumeration of multiple objects^3^ and recent findings on enumeration of object parts^26^.

The EEG results provide novel information about the neuronal correlates of multi-part enumeration and subitizing. First, in line with previous studies on separate objects we found that the N2pc was modulated as a function of the numerosity of object parts in both Experiment 1 and 2. Thus, paralleling the case of object individuation^7^,^21^ this effect indicates that multiple parts of a single object can be individuated simultaneously. Second, just as in the case of neurophysiological studies on enumeration of multiple objects^18^,^21^,^22^, the N2pc modulation in enumerating multiple object parts reported here reached an asymptote at 3-4 target elements. These results reinforce the view that this neural response is sensitive to the numerosity of both objects and object parts. Crucially, the results also show that the subitizing limits at the behavioural and neurophysiological levels are interconnected, as indicated by the fact that the N2pc tracks the magnitude of the behavioural subitizing limit (i.e., three targets in Experiment 1; four targets in Experiment 2). That is, the behavioural and N2pc functions both displayed an elbow pattern, and the two patterns co-varied in the two experiments reported here. Thus, the N2pc limit follows the variation in (behavioural) subitizing limit, rather than reaching a plateau at a fixed numerosity. This further highlights that the neurophysiological mechanism underlying the generation of N2pc crucially supports the individuation of multiple parts of objects.

Our findings resonate with the results of previous EEG studies using distinct objects, where the N2pc component has been shown to increase with the increase in the number of target elements to-be-enumerated^19^,^20^,^21^ or tracked^18^, reaching an asymptote at approximately 3-4 target objects. In our study, we did not directly compare object parts with disconnected objects. While this aspect may represent a potential limitation of the present experiments, it is important to note that the N2pc pattern found here closely resembles those found in previous studies on multiple object processing. The similarity suggests that the visual system can isolate objects or object parts with the same efficiency, and invites the inference that parts of an object are individuated by the same neuronal mechanism as the one individuating distinct objects. This implies that individuation can flexibly operate over separate objects as well as separate elements connected into a single object.

The difference in the behavioural and neural limits between the two experiments could be accounted for by several factors related to physical parameters, in addition to the intrinsic variability in the two groups of participants (individual differences are known to affect the subitizing limit^20^,^21^,^22^). For example, it has previously been shown that increasing stimulus eccentricity decreases enumeration performance^32^. This aspect could explain why performance was lower in Experiment 1, where the parts to be enumerated were further away from the centre, than in Experiment 2, where the parts were closer to the centre. However, the differences between the two experimental conditions could also be driven by many other factors. Thus, further research is required to evaluate which factors influence performance in enumerating parts of an object.

In our study, the increase in the number of parts was confounded with an increase in other variables, such as luminance. One may wonder whether the N2pc effect that we observed in our study reflects a change in low-level factors rather than the individuation of object parts. Earlier studies have shown that the N2pc is not modulated by object numerosity when object numerosity is not task-relevant^33^. In addition, N2pc is always modulated by target numerosity irrespective of the total variation in the number of distracters (which represents a large physical change, see ref. 20). These results indicate that variation in physical parameters cannot be a good account for the N2pc numerosity-related pattern. Thus, together with previous findings showing that the N2pc indexes the deployment of attention^17^ and that its amplitudes vary with the number of relevant objects^19^,^20^, we suggest that N2pc also reflects the individuation of object parts.

It could be argued that N2pc does not reflect an object individuation mechanism but a numerical representation of the number of objects (e.g. the concept of “1”). However, N2pc has traditionally been observed with non-numerical stimuli in visual search tasks^34^,^35^. This suggests that this neural response is not specifically tied to numerical representation. Furthermore, it has been shown^36^ that N2pc amplitude increases with the increase in the number of Arabic numerals to-be-enumerated, irrespective of the identity of these numerals. Thus, N2pc seems to reflect the individuation of a small number of objects rather than numerals. Finally, the similarity in the N2pc patterns between enumeration and other tasks (such as multiple object tracking paradigms^18^) suggests that the N2pc reflects a mechanism of efficient individuation of approximately 3-4 elements that is used in tasks requiring multiple object processing. Together, these results indicate that individuation, as reflected by N2pc, and numerical representation, recruit distinct neuronal mechanisms.

The N2pc has traditionally been measured in visual search contexts^17^,^34^ and was originally interpreted as a reflection of distractor filtering^37^,^38^, although an alternative explanation in terms of target processing/enhancement has also been proposed^39^,^40^,^41^. The numerosity-related changes in N2pc amplitudes^18^,^21^,^33^ has further promoted the proposal that this response may reflect a feature-to-location binding mechanism that can be applied simultaneously to a limited set of elements, leading to their efficient individuation^23^. Within this explanatory framework, the present results on object parts can easily be accommodated by assuming that the indents or outdents of the stimuli used here represent distinct features, which can individually be bound to a specific location – that is, individuated. It should be further noted that, here, we measured a numerosity-related modulation of N2pc without distracting elements in the relevant hemifield (i.e., the hemifield containing the target parts), further undermining an interpretation of this EEG response as exclusively reflecting distractor suppression.

An MEG study^42^, specifically designed to localize the source of the N2pc, has shown that this component is composed of an early neural response in the parietal areas and a later occipito-temporal response. These areas are known to be tuned to object quantities in humans^43^,^44^,^45^ as well as in other animal species^46^,^47^. Our data, showing that individuation of object parts is reflected by the N2pc component, extend this view by suggesting that within-object quantity is also efficiently coded by these areas. Thus, integrating the results of this study with the ones from previous research on multiple object processing, allows us to argue for the existence of a single, attention-based individuation mechanism that flexibly operates over separate objects or object parts, making available a representation of a limited set of individual elements that is appropriate for exact enumeration.

The novel findings reported here offer an exciting tool for addressing issues related to the role of objecthood and spatial uniqueness in the individuation process, for at least two reasons. First, not all previous studies have shown the occurrence of subitizing for separate features of the same object^4^, but the exact visual cues that allow for efficient individuation and accurate performance are still unknown. For instance, connectivity could be the key factor that allows parts of a single object to maintain spatial distinctiveness^26^. While recent studies^48^,^49^ have started to investigate this aspect on numerosity estimation, the effect of connectivity on exact enumeration has not systematically been addressed. Nonetheless, in line with previous studies^26^, our results suggest that connectivity does not eliminate the subitizing effect. Second, our results indicate that object parts are individuated at around the same time (approximately from 180 ms post-stimulus) as distinct objects. This may suggest that the information about the location of both the object and its parts is computed simultaneously, or that parts of an object are individuated first because of the task requirements (and subsequently bound into one single object representation). Assessing the time course of the neural activity related to variation in object parsing, and testing for the presence of the N2pc numerosity-related pattern could provide new insight on these issues, and allow for objective measurements in setting the boundaries between objecthood and spatial distinctiveness.

Finally, the results on the CDA amplitude indicated a modulation as a function of the number of parts to be enumerated, as had been found in previous studies on object enumeration and multiple object tracking^18^,^22^. However, in contrast with the N2pc, the CDA pattern did not track the subitizing limit: in both experiments the CDA reached an asymptote at a fixed target numerosity (four target parts), despite the fact that the performance limit was different across the two experiments (see also Supplementary Data). This result is consistent with previous studies on N2pc and subitizing in enumerating separate objects^21^,^22^, which indicate that enumeration abilities correlate better with the functioning of the mechanism reflected in the N2pc than in the CDA. CDA amplitudes are sensitive to several factors, such as the number of object representations in visual working memory^27^,^28^ and task requirements^50^,^51^,^52^. Previous results have also pointed to the role of object complexity in the modulation of CDA^53^,^54^ (but see also refs 55, 56, 57). Thus, it could be argued that the effect found in the present study is not strictly driven by the number of parts, but rather by the fact that increasing part numerosity correlated with an increase in object complexity. Further research is required to determine the role of object complexity in the individuation of multiple parts of a single object.

In conclusion, our study showed the presence of a subitizing effect for up to 3-4 parts of an object. This effect was mirrored by a modulation of N2pc amplitudes up to 3-4 items. Given the similarity with previous results on distinct objects^19^,^21^, these findings invite the inference that the same individuation mechanism operates on multiple objects and multiple parts of a single object.

## Methods

### Participants

Fourteen participants were tested in Experiment 1 (all women, 1 left-handed, age 24 ± 4 *MEAN* ±*STD*) and in Experiment 2 (12 women, 1 left-handed, age 23 ± 4). Two additional participants in Experiment 1 and one in Experiment 2 were excluded from further analyses because of excessively noisy EEG signal. None of the participants tested in Experiment 1 participated in Experiment 2. All participants had normal or corrected to normal acuity and provided written informed consent. They received monetary compensation or course credit for their participation. The study was conducted in accordance with the Declaration of Helsinki and approved by the University of Trento Ethics Committee.

### Stimuli

The stimuli of Experiment 1 consisted of two solid half discs, one in each hemifield (see Fig. 1) presented on a grey background (23 cd/m2, x = 0.28, y = 0.31). One of the half discs was green and the other one was orange. The centre of each half disc was located at 3° visual angle from the centre of the screen with a radius of 6° visual angle. In Experiment 2 the stimulus was a grey circle (23 cd/m2, x = 0.28, y = 0.31) presented at the centre of the screen with a radius of 6° visual angle. The screen background during the stimulus presentation was divided into two colours: one side was green and the other side was orange (see Fig. 1). The screen background presented during fixation and response periods was from a darker grey than the stimulus (9.1 cd/m2, x = 0.28, y = 0.31).

**Figure 4:**
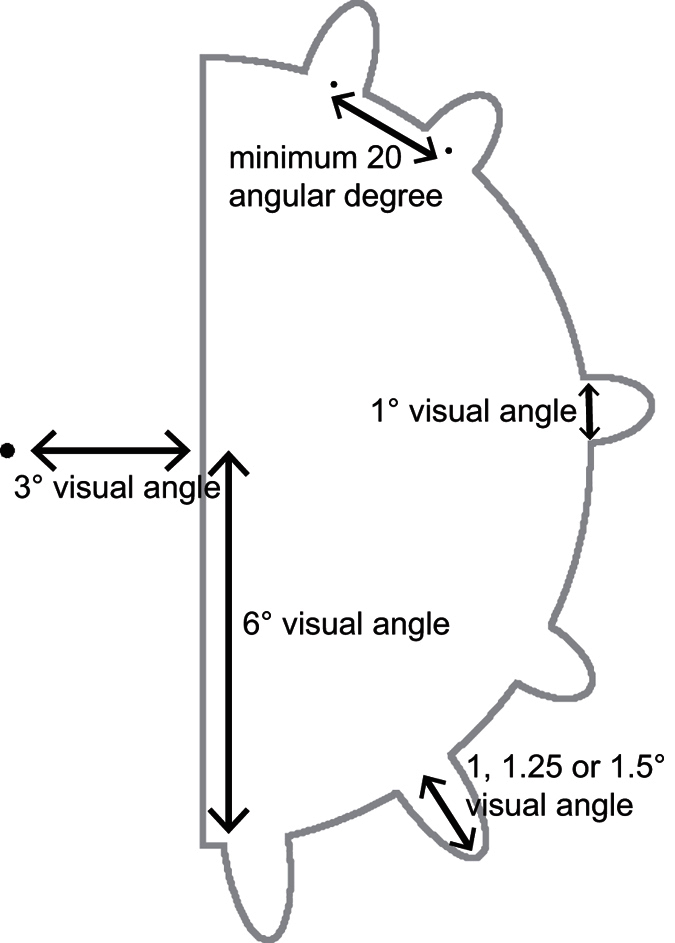
Description of the stimulus in Experiment 1. The same parameters were used to draw the indents in Experiment 2.

In both experiments, a variable number of parts (outdents in Experiment 1 and indents in Experiment 2) from 1 to 6 was presented in each hemifield. The location of each part was randomly chosen between 7 to 172 angular degrees from the vertical with a minimum of 20 angular degrees separating the centre of adjacent parts (see Fig. 4). The part length was 1° visual angle and its height was 1, 1.25 or 1.5° visual angle. The total number (but for catch trials, see procedure below) and heights of the parts were the same in both hemifields but their specific locations were not. This ensured that any lateralized differences in the EEG signal could not be explained by a lateralized difference in the stimuli properties.

The green and orange colours were set to be equiluminant for each participant using the standard heterochromatic flicker procedure. In this procedure, performed prior to the experimental session, a square flickering between a green and an orange colour was presented on the screen. By pressing the left or right arrow key, each participant could adjust (increase or decrease) the amount of green until the square would flicker the least (the orange value was fixed at 8.8 cd/m2, x = 0.52, y = 0.42). This procedure was repeated 8 times; the average green value was then used in the experiment (on average for all participants the green value was 9.68 cd/m2, x = 0.29, y = 0.57; minimum 8.3 cd/m2; maximum 10.8 cd/m2).

### Procedure

Participants were seated in a dimly room at approximately 75 cm from a CRT screen (1024 × 768, screen length = 32 cm, refresh rate = 100 Hz, head position was unconstrained). The experiment was run using MATLAB (Mathworks) and the Psychtoolbox^58^,^59^. Participants were asked to enumerate as accurately as possible the number of parts of an object (outdents in Experiment 1, indents in Experiment 2) located on the cued side of the screen. The cue was a colour, green during half of the experiment and orange during the other half of the experiment (the order was counterbalance across participants), indicated by either the object itself in Experiment 1 or by the background in Experiment 2.

At the beginning of each trial, a fixation point (subtending 0.4° visual angle) was presented at the centre of a grey screen for a random duration between 1000 and 1300 ms (see Fig. 1). The stimulus was presented for 150 ms. 800 ms after stimulus offset, a response screen with the possible answers appeared (digits 1,2,3,4,5 and 6). Participants provided their response using the mouse by clicking on one of the digits. Trials with no response within 4 sec. were excluded from the analysis (0.07% in Experiment 1, 0.19% in Experiment 2). Participants were asked to keep their eyes on the fixation point until the response screen was presented.

The experimental session consisted of 20 blocks of 60 trials (10 trials per numerosity, equally balanced between left and right side target presentation). In each block, three additional catch trials were included in which the number of parts was unequal between the target and the distractor side. These catch trials were not included in the analyses given their small number, but they ensured that participants attended to the target side. Feedback on accuracy performance for each numerosity was displayed at the end of each block.

### Behavioural analysis

To estimate the subitizing point in the two experiments, we used two procedures. In one procedure, each participant’s ER from 1 to 6 numerosities was fitted to a standard exponential function (eq. 1):


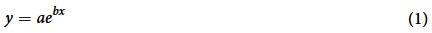


We then computed the point at which the fitted curve reached 8% of errors and considered that value as the subitizing span for that participant (this 8% threshold is arbitrary as any threshold between 5 and 10% can be considered as efficient enumeration; changing this threshold might produce slightly different subitizing span values, which are reported in the Supplementary Data, but any relationship with EEG components or any differences between the two experiments would remain the same). In the second procedure, each participant’s ER was fitted to a bilinear curve with the first slope fixed at 0^21^. For both methods, we also calculated each individual adjusted r^2^ to quantify how well the model explained each participant’s ER. Note that given the relatively limited range of numerosity tested in this study, there is a risk of data overfitting, particularly with the bilinear model. The subitizing span value returned by both methods should therefore only be taken as a general indication.

### EEG recording and data analyses

EEG activity was recorded from a standard-10-5 system with 60 Ag/AgCl electrodes cap (EasyCap, Brain Products, Germany) at a sampling rate of 1 kHz and a high-pass filter of 0.01 Hz. AFz was used as the ground and the right mastoid was used as reference. Four additional electrodes were used: one was placed on the left mastoid, one below the right eye to record eye-blinks, and two were placed at the outer canthi of both eyes to record horizontal eye-movements. Impedance was kept below 10 kΩ for all electrodes. The signal was re-referenced off-line to the average of the left and right mastoids. Data pre-processing was conducted using EEGLAB^60^. The EEG signal was low-pass filtered at 40 Hz and divided into epochs from −200 ms to 600 ms relative to stimulus onset. Each epoch was baseline corrected by subtracting the average activity between −200 ms to 0 ms for each electrode. Two different procedures were used for artefact rejection. In the “visual” procedure, epochs with muscle artefacts, blinks and horizontal eye-movements were rejected after visual inspection (on average, 10% of trials were rejected in Experiment 1, 7% in Experiment 2). In the “automatic” procedure, epochs with any electrode amplitude exceeding ± 80 μV or with a difference exceeding ± 30 μV between the left and right eye-movement amplitude were discarded from the analysis. Following this automatic procedure, two participants with more than 30% of rejected trials were excluded in Experiment 1 and one in Experiment 2. On average, the proportion of rejected trials for the remaining participants was 15% in Experiment 1 and 10% in Experiment 2.

For each participant, we calculated the average EEG activity considering correct responses for each target presentation side (left or right) and each target numerosity from 1 to 5. Numerosity 6 was not included because of the low number of correct responses. Based on previous EEG enumeration studies^19^,^21^,^22^, EEG analysis were performed on all parieto-occipital electrodes available (PO7, PO8, PO3, PO4, O1, O2). The lateralized EEG amplitude was computed by subtracting the EEG amplitude of each electrode located in the ipsilateral hemifield relative to the target (e.g. PO7, for a target in the left visual field; PO8, for a target in the right visual field) from the EEG amplitude of each electrode located in the contralateral hemifield relative to the target. For each participant the EEG signal was collapsed across target side and averaged within two time-windows corresponding to the N2pc and the CDA components. The N2pc was measured in the 180–250 ms range post-stimulus onset. This time window was determined by visual inspection of the data (and was mainly driven by the fact that the positive rebound that often follows the N2pc was unexpectedly larger and slightly earlier than expected from previous findings). For the CDA component, we considered a time-window between 400–600 ms, similar to what has been used in previous studies^22^. Finally, the signal was averaged over all the parieto-occipital electrodes.

To test for an effect of numerosity, analyses were conducted using repeated-measures (RM) ANOVA with numerosity (1–5) as main factor. Greenhouse-Geisser correction was applied when necessary. From previous studies^19^,^21^, we expected the N2pc amplitude to reach an asymptote at around the subitizing point (3-4 elements). To get an estimate of the asymptote, we conducted post-hoc analyses by means of Helmert contrasts.

## Additional Information

**How to cite this article**: Poncet, M. *et al*. Individuation of objects and object parts rely on the same neuronal mechanism. *Sci. Rep.* **6**, 38434; doi: 10.1038/srep38434 (2016).

**Publisher’s note:** Springer Nature remains neutral with regard to jurisdictional claims in published maps and institutional affiliations.

## Supplementary Material

Supplementary Data
