# Supplementary material for: Individuation of objects and object parts rely on the same neuronal mechanism
Source: Sci Rep. 2016 Dec 7;6:38434. doi: 10.1038/srep38434 (PMC5141436; doi:10.1038/srep38434)
Supplement: Supplementary Data [file srep38434-s1.pdf]

Individuation of objects and object parts rely on the same neuronal mechanism

Marlene Poncet, Alfonso Caramazza, Veronica Mazza

Supplementary Data

Subitizing span

In our study, we estimated the subitizing point using an exponential function and computed the point at which the fitted curve reached 8% of errors. As mentioned in the method section, this 8% threshold is arbitrary and any value between 5 and 10 % can be considered as efficient enumeration. If we consider the point at which the exponential curve reached 5% of errors, the subitizing span is 2.25± 0.29 in Experiment 1 and 3.71±0.24 in Experiment 2. Considering a 10% threshold, the subitizing span is 3.32±0.25 in Experiment 1 and 4.50±0.17 in Experiment 2.

Results based on automatic artefact rejection procedure

- **N2pc**

In Experiment 1, we observed a significant effect of numerosity on N2pc amplitudes ($F(4,44)=3.10$, $p=0.02$, ${}_{p}\eta^2=0.22$) and significant Helmert contrasts for numerosities 1 and 2 ($F(1,11)=7.32$, $p=0.02$, ${}_{p}\eta^2=0.40$ and $F(1,11)=6.20$, $p=0.03$, ${}_{p}\eta^2=0.36$, respectively) but not for larger numerosities ($p>0.6$).

In Experiment 2, we observed a main effect of numerosity ($F(4,48)=14.11$, $p=1x10^{-7}$, ${}_{p}\eta^2=0.54$). N2pc increased between 1- and 4-parts objects ($F(1,12)=48.06$, $p=2x10^{-5}$, ${}_{p}\eta^2=0.80$; $F(1,12)=14.00$, $p=0.003$, ${}_{p}\eta^2=0.54$; $F(1,12)=3.29$, $p=0.09$, ${}_{p}\eta^2=0.21$; for each Helmert contrast respectively) but there was no difference in amplitude when 4 or 5 parts were presented ($F(1,12)=0.0004$, $p=0.98$).

- **CDA**

In Experiment 1, a significant effect of numerosity was observed on CDA amplitude ($F(1.82,20.02)=22.69$, $p=1x10^{-5}$, ${}_{p}\eta^2=0.67$). Helmert contrasts were all significant up to 4 parts ($p<0.05$ for each contrast) but not for the contrast between 4 and 5 parts ($F(1,11)=0.13$, $p=0.73$).

In Experiment 2, we found a main effect of numerosity on CDA ($F(2.29,27.48)=60.79$, $p=3x10^{-11}$, ${}_{p}\eta^2=0.83$) with significant Helmert contrasts up to the numerosity 4 ($p<0.001$ for each contrast) but no difference between numerosities 4 and 5 ($F(1,12)=1.82$, $p=0.20$).

Comparison between N2pc and CDA components

In our results, target numerosity seems to influence N2pc and CDA in different ways. To substantiate this observation, we conducted a RM-ANOVA with numerosity and component as main factors. In both experiments, we found a significant interaction between numerosity and component ($F(2.16,28.03)=13.33$, $p<10-5$, ${}_{p}\eta^2=0.51$; $F(4,52)=41.07$, $p=2x10-15$, ${}_{p}\eta^2=0.76$; for Experiment 1 and 2 respectively), indicating that the numerosity-related patterns for the N2pc and CDA components are different from each other.
